# Chlorosis

**Published:** 1844-06

**Authors:** 


*Chlorosis.*—Chlorosis, according to our view of its pathology, is *accompanied*, but not *induced* by the absence of the catamenia; and hence it seems to us that we may, with perfect safety, keep the circumstance of the amenorrhœa almost out of consideration, in conducting its treatment. The disease is essentially a disease of the circulating fluids, and may, it is well known, exist in the male sex as well, though not so frequently, as in the female. There is a deficiency of the red globules of the blood, and an excess of its serum. If we are to trust the recent researches of M. Andral—and his authority, we need not say, is not lightly to be disputed—the proportion of the red globules in chlorotic blood is less by one-half, or even two-thirds, than the normal quantity. Here is the "fons et origo mali:" we have a poor watery state of the circulating fluid, which nature finds insufficient for the purposes of healthy nutrition and secretion—at that very period of life, when these functions ought to be the most energetic. Although we could by the use of any means provoke the catamenial secretion under such circumstances, would it, think you, have any beneficial effect upon the general health?—we judge not. Menstruation, be it remembered, in one sex has its analogous function (although this is not periodic) in the other; and certainly no one would ever think of administering any aphrodisiac medicines to a chlorotic boy, for the cure of his green-sickness. Let but the system have once a due supply of heathy blood permeating all its parts, and stimulating all its organs, and then will the white-waxen look give way to a rosy freshness, the faded and capricious appetite to a natural relish for wholesome food, the peevish listlessness and inaction to sprightly animation and vigor, and, with these changes, the absence of the menstrual secretion will be replaced by a due and normal regularity. Such will be the effects of a judicious hygienic treatment by steel and other tonics, by exercise in the open air, and a generous diet—not forgetting Falstaff's sovereign remedy for male green-sickness. "The second property," says the old rogue, "of your excellent Sherries is the warming of the blood; which, before, cold and settled, left the liver white and pale, which is the badge of pusillanimity and cowardice; but the Sherries warms it, and makes its course from the inwards to the parts extreme; it illuminateth the face, which, as a beacon, gives warning to all the rest of this little kingdom, man, to arm; and then the vital commoners and inland petty spirits muster me all to their captain, the heart; who great, and puffed up with this retinue, doth any deed of courage."—*Med. Chi. Rev.*, Jan.

